# Editorial: Iron Nutrition and Interactions in Plants

**DOI:** 10.3389/fpls.2019.01670

**Published:** 2020-01-10

**Authors:** Wolfgang Schmidt, Sebastien Thomine, Thomas J. Buckhout

**Affiliations:** ^1^ Institute for Plant and Microbial Biology, Taipei, Taiwan; ^2^ Institute de Biologie Intégrative de la Cellule (I2BC), Gif-sur-Yvette, France; ^3^ Institute of Biology, Humboldt University of Berlin, Berlin, Germany

**Keywords:** iron, iron homeostasis, iron transport, plant mineral nutrition, bio-fortification, soil biology, regulation of gene expression

*Gold is for the mistress—silver for the maid—*

*Copper for the craftsman cunning at his trade!*

*“Good!” said the Baron, sitting in his hall,*

*“but iron—cold iron—is master of them all.”*

Rudyard Kipling

Iron is a central component of electron chains and a co-factor of many vital enzymes. Only a few bacteria are able to substitute iron with other metals, making it an essential element for virtually all life forms. In plants, iron is also required for photosynthesis and chlorophyll synthesis. The availability of iron in soils dictates the distribution of plant species in natural ecosystems and limits yield and nutritional quality of crops. Insufficient iron uptake causes retarded growth, interveinal chlorosis, and reduced fitness. Sufficient iron levels in food crops are critical to combat iron deficiency-induced anemia, one of the largest nutritional disorder worldwide. Too much iron is, however, toxic to cells. It is therefore mandatory for plants to overcome the often-restricted availability of soil iron by strategies that increase its mobility and restrict its uptake when present in excess.

Despite great leaps forward in the research into plant iron nutrition over the past decades, many facets of cellular iron homeostasis still await further clarification. Moreover, attempts to increase the iron content in edible plant parts are far from having reached sufficient improvement in dietary iron intake. The International Symposium of Iron Nutrition and Interaction in Plants (ISINIP) is a biannual meeting that covers a wide range of aspects, including but not limited to iron availability in the soil, the regulation of cellular iron homeostasis, and the exploration of novel avenues for fortifying plants with iron. The collection of mini reviews, perspectives, and original papers presented in this Research Topic is in part associated with contributions to the 19^th^ edition of the ISINIP held in 2018 in Taipei, Taiwan.

## Checkpoints, Barriers, and a Minefield of Inadvertent Interactions: The Journey of Iron Through the Cell

While the mechanisms underlying the uptake of iron from the soil are relatively well understood, the trafficking of iron to chloroplasts and mitochondria is less well explored. Chloroplasts are loaded with transition metals and represent the iron-richest system in plant cells. Research conducted by the group of Kathrin Philipar suggested that the ATP-binding ABC-transporter subunits ABCI10 and ABCI11 are part of a novel module of a prokaryote-type ECF/ABC transporter with similarities to components of prokaryotic multi-subunit ABC transporters putatively involved in metal ion uptake (Von Voithenberg et al.). New transporters have been identified also for the transport of iron through the inner mitochondrial membrane. Jain et al. show that the Arabidopsis transporters MIT1 and MIT2 are involved in iron import into mitochondria and critical for mitochondrial function.

Iron is highly reactive and must be chelated throughout intra- and intercellular trafficking to avoid cellular damage. Nozoye et al. are reporting a collaborative approach to elucidate the function of the nicotianamine (NA) transporter EFFLUX TRANSPORTER OF NA (ENA1) in rice, which is involved in the transport of NA to the apoplast and in the import of iron into cellular compartments such as plastids. The authors conclude that in rice NA transport by ENA is critical for cellular iron homeostasis by shielding iron ions from interacting with other molecules, causing precipitation of iron and oxidative damage to the cell.

## All for One: A Multitude of Regulators Control Cellular Iron Homeostasis

The regulation of iron uptake has to meet, but not to exceed the demand of the plant to avoid undernourishment or toxicity. Therefore, cellular iron levels are controlled by a delicate signaling network that orchestrates the demand of different plant parts in the context of rapidly and constantly changing availability of iron in the soil. A key role in the regulation of iron uptake is played by bHLH proteins. Until now, 16 bHLH transcription factors of this type were shown to be involved in the control of cellular iron homeostasis; it can be expected that this number is not yet exhaustive. In their mini review, Gao et al. summarize the current knowledge regarding the complex network of bHLH proteins that cooperatively regulate iron uptake in the green lineage. The bHLH protein FIT (bHLH29) takes a central position in this regulatory network. The activity of FIT is sophisticatedly controlled by a suite of proteins, which either activate FIT or enhance its degradation. Wu and Ling review the action of proteins that have been shown to interact with FIT and post-transcriptionally fine-tune its activity.

While the mechanisms underlying the perception of the cellular iron status differs across the kingdoms of life, some actors involved in iron sensing appear to be commonly recruited by yeast, mammals, and plants. Glutaredoxins and partnering BOLAs, homologs of *Escherichia coli* BolA proteins, may represent such common actors. In their Perspective paper, Rey et al. speculate that glutaredoxins, alone or in complex with BOLAs, may function in the maintenance of iron homeostasis in plants. More evidence for a specific function in iron signaling has accumulated for a small family of hemerythrin E3 ubiquitin ligases unique to the green lineage. HRZ (Hemerythrin RING Zinc finger) proteins in rice and their homologs BTS (BRUTUS) and BTSL (BRUTUS-LIKE) in Arabidopsis modulate the activity of specific transcription factors in a negative feedback loop, and are critical in avoiding the uptake of toxic amounts of iron. In their mini review, Rodríguez Celma et al. provide an overview on what is known about the putative function in iron sensing and signaling of this enigmatic group of regulators.

Plant growth-promoting bacteria can trigger both induced systemic resistance (ISR) and a partial iron deficiency response, indicating overlap in the regulation of the two processes. Infection with growth-promoting bacteria improves plant growth, increases iron content, and boosts plant defenses, conferring resistance to pathogens and pests. Romera et al. review the available data on this “ironic liaison,” and investigate the possibilities to use ISR-eliciting microbes as both pesticides and iron fertilizers in a more sustainable agriculture.

Kaisalam et al. took a different approach toward the dissection of iron signaling pathways. The authors identified two small molecules, referred to as R3 and R6, through a chemical screen and showed that both molecules efficiently compromised the iron deficiency response of Arabidopsis, likely by targeting different signaling pathways. While R6 influences the IVc clade of bHLH proteins, R3 appears to affect a so far unexplored route toward the transcription factor FIT.

Post-translational modifications of lysine residues of core histones such as acetylation or methylation are a key component of eukaryotic gene regulation. Park et al. proposed that PRC2 (Polycomb Repressive Complex 2)-mediated methylation of lysine 27 of histone 3 attenuates the induction of FIT target genes, restricting iron uptake to avoid possible overload. The authors conclude that this epigenetic feature prevents maximum induction of genes involved in iron acquisition, providing an additional mechanism to fine-tune gene activity.

MicroRNAs (miRs) are a further node in the iron-signaling network. Carrió-Seguí et al. show that miR408 targets laccase-like multicopper oxidases with putative promiscuous activities interfering with the redox homeostasis and, ultimately, with iron signaling. The study further show that modifying miR408 levels resulted in complex phenotypes characterized by increased oxidative stress, altered lignification and, as a consequence of the latter, compromised metal transport.

## *Liaisons Dangereuses*: Interaction of Iron With Other Minerals

The iron homeostasis network is sophisticatedly interwoven with the uptake and transport of other mineral nutrients, often in an antagonistic manner. Lucena et al. addressed such interactions between iron and phosphate and found that deficiencies in one nutrient resulted in transitory induction of the expression of genes encoding proteins involved in the uptake of the other. The authors speculate that this induction is possibly triggered by phloem signals originating in the shoots that interact with hormones to balance the uptake of mineral nutrients from the soil. An antagonistic crosstalk between iron and phosphate signaling was also reported with respect to the accumulation of catecholic coumarins (Chutia et al.). The authors show that phosphate availability directly influences iron deficiency-induced accumulation of coumarins, a process that appears to reflect crosstalk between the regulators controlling the responses to iron and phosphate deficiency.

Similar to what have been previously reported for strategy I plants, Nicolic et al. observed an ameliorative effect of silicon on iron deficiency stress in barley, a plant with predominant Strategy II iron acquisition. Silicon significantly modulated the expression of Strategy II genes and increased the total iron content in the youngest leaves, which was associated with a lower ROS level, elevated APX and CAT activities, and increased chlorophyll levels. Moreover, silicon improved root-to-shoot iron translocation, resulting in an increased iron content in the youngest leaves.

Studying environmental cues such as Fe deficiency in isolation covers the processes that orchestrate the hierarchy of responses to simultaneously perceived signals and stresses. Terri Long’s group developed an image analysis pipeline to extract spatiotemporal metrics of GFP signals showed that subjecting the plants to both heat stress and iron deficiency affected the spatiotemporal function of the mitosis marker CYCB1;1 antagonistically, suggesting interplay between stress pathways when plants are exposed to multiple environmental factors that may modulate the response that is observed in experiments that singles out a specific stimulus (Buckner et al.).

## Too Much to Handle: Strategies to Avoid Iron Toxicity

If in excess, iron can react with hydrogen peroxide and trigger the formation of harmful hydroxyl radicals through Fenton chemistry. The free iron concentration drastically increases with decreasing redox potential, as it is the case for instance in flooded soils. Although genetic variation in iron tolerance suggests the evolution of adaptive strategies to cope with high external iron levels, the mechanisms underlying such adaptations have not yet been fully elucidated. Stein et al. speculated that natural variation in iron toxicity tolerance across rice cultivars is related to the composition of the root cell wall. The authors demonstrate that increased lignification in the outer layers of the cortex and in the vascular bundle may alter iron permeability, radial diffusion, and root-to-shoot translocation of iron, ultimately leading to a higher tolerance to high external iron levels. Also the interaction of iron with other nutrients appears to be important for coping with high iron levels. Wu et al. reported that the inward potassium ion (K^+^) channel OsAKT1 affects the translocation of iron from roots to shoots, a process that is contributing to the tolerance to iron toxicity. Loss-of-function mutants of OsAKT1 exhibit altered K homeostasis, lower K^+^ concentrations, and hyperpolarization of the plasma membrane, leading to decreased iron loading into the xylem. A further important player in the tolerance of rice to high iron levels is the vacuolar iron reductase OsFRO1, which maintains cellular iron homeostasis by tuning the iron concentrations in the cytoplasm and in the vacuole (Li et al.).

The metal chelator NA plays multifaceted roles in metal homeostasis and appears also to be important to protect the plant from iron overload. Aung et al. show that expression of the rice NA synthase *OsNAS3* strongly increased when plants were subjected to excess iron levels; *OsNAS3* knockout plants showed reduced growth and severe leaf bronzing, resulting from increased iron accumulation. The results demonstrate that NA is important for mitigating excess iron and provide a new perspective for the development of rice lines with improved tolerance to excess iron availability soils.

## The More, the Better? Fortification of Plants With Iron

More than 30% of the arable land are calcareous and potential iron-deficient. Low iron content of crops is the main cause of iron deficiency-induced anemia, the most common micronutrient deficiency globally. Iron-biofortification denotes the process of enhancing bioavailable iron in edible parts of staple food by agronomic approaches, biotechnology techniques, or conventional plant breeding. Biofortification is a promising approach to combat micronutrient malnutrition, which affects nearly one-third of the world’s population, particularly in resource-limited settings.

Iron fertilizers are costly, not environmentally friendly, and often inefficient. Iron-humic complexes have long been known to promote iron nutrition in a multifaceted way by providing a readily available iron form in the soil and by directly impacting physiological and developmental programs. Zanin et al. review the complex issue of how humic substances affect plant performance and investigate the possibility to use iron-humic complexes as an environmentally friendly source of iron fertilizer. The supposition that increasing crop production through innovative iron fertilizers is a promising alternative to traditional iron fertilization is also supported by a study by Cieschi et al. The authors describe the action of three iron-humic nanofertilizers as a natural, low cost and environmental option to improve iron uptake of soybean grown on calcareous soils by providing iron over prolonged time periods.

Intercropping of fruit trees or crops with graminaceous plants can have beneficial effects on the iron status of iron-inefficient plants and has been proven useful for low-input/resource-limited agricultural systems. Dai et al. review the effects and mechanisms underlying the promotive effects on iron nutrition focusing on Leguminosae/Gramineae intercropping systems. Intercropping is particularly beneficial for calcifuge species adapted to acidic soils. Blueberry, a calcifuge, develops severe iron deficiency symptoms under alkaline conditions, drastically reducing plant growth and yield. Intercropping with the grasses *Festuca rubra* and *Poa pratensis* in combination with Fe-EDDHA applications was found to be effective in increasing fruit load and yield of blueberries while reducing the skin/flesh ratio and firmness of the berries (Michel et al.).

Genetic biofortification, i.e. conventional breeding and genetic engineering, is the topic of a mini review by Ludwig and Slamet-Loedin. The authors summarize the problems and advances of different biofortification strategies to enrich rice and wheat grain iron to combat “hidden hunger.” Rice has employed components of both strategy I and strategy II to acquire iron from the soil. Masuda et al. reported that transgenic rice with increased phytosiderophore production and Fe(III) reduction activity conferred by introducing a gene cassette comprising a regulator of the iron deficiency responses (IRO2), a barley IDS3 genome fragment to increase PS production, and a mutationally reconstructed yeast ferric reductase exhibited enhanced tolerance to iron-deficient conditions and significantly higher yield when cultivated on calcareous soil.

The distribution of iron in seeds in a main determinant for its bioavailability, which can be severely restricted by antinutrients such as phytate that are localized in the same cellular compartments. With the goal to investigate if iron can be stored in phytate-free compartment such as plastids, Eroglu et al. investigated metal localization in seeds of more than twenty species using histochemical or X-ray based techniques. The study revealed distinct seed iron storage patterns across plant lineages. The authors further report that in Rosids iron is concentrated in the innermost cell layers, the endodermis, and in the cortex.

## Author Contributions

All authors wrote and approved the manuscript.

## Conflict of Interest

The authors declare that the research was conducted in the absence of any commercial or financial relationships that could be construed as a potential conflict of interest.

